# Predicting segregation of multiple fruit-quality traits by using accumulated phenotypic records in citrus breeding

**DOI:** 10.1371/journal.pone.0202341

**Published:** 2018-08-16

**Authors:** Atsushi Imai, Takeshi Kuniga, Terutaka Yoshioka, Keisuke Nonaka, Nobuhito Mitani, Hiroshi Fukamachi, Naofumi Hiehata, Masashi Yamamoto, Takeshi Hayashi

**Affiliations:** 1 Institute of Fruit Tree and Tea Science, National Agriculture and Food Research Organization, Fujimoto, Tsukuba, Ibaraki, Japan; 2 Graduate School of Life and Environmental Science, University of Tsukuba, Tennodai, Tsukuba, Ibaraki, Japan; 3 Western Region Agricultural Research Center, National Agriculture and Food Research Organization, Senyucho, Zentsuji, Kagawa, Japan; 4 Institute of Fruit Tree and Tea Science, National Agriculture and Food Research Organization, Okitsunakacho, Shimizu, Shizuoka, Japan; 5 Kenou Development Bureau, Nagasaki Prefectural Government, Eishohigashimachi, Isahaya, Nagasaki, Japan; 6 Faculty of Agriculture, Kagoshima University, Korimoto, Kagoshima, Kagoshima, Japan; 7 Institute of Crop Science, National Agriculture and Food Research Organization, Kannondai, Tsukuba, Ibaraki, Japan; United States Department of Agriculture, UNITED STATES

## Abstract

In the breeding of citrus (*Citrus* spp.), suitable fruit quality is essential for consumer acceptance of new cultivars. To identify parental combinations producing F_1_ progeny with fruit-quality traits exceeding certain selection criteria, we developed a simple and practical method for predicting multiple-trait segregation in an F_1_ progeny population. This method uses breeding values of parental genotypes and an additive genetic (co)variance matrix calculated by the best linear unbiased prediction method to construct a model for trait segregation in F_1_ progeny. To confirm the validity of our proposed method, we calculated the breeding values and additive genetic (co)variances based on phenotypic records on nine fruit-quality traits in 2122 genotypes, and constructed a trait segregation model. Subsequently, we applied the trait segregation model to all pairs of the 2122 genotypes (i.e., 2,252,503 combinations), and predicted the most promising combinations and evaluated their probabilities of producing superior genotypes exceeding the nine fruit-quality traits of satsuma mandarin (*Citrus unshiu* Marcow.) or ‘Shiranuhi’ (‘Kiyomi’ × ‘Nakano No. 3’ ponkan), two popular citrus cultivars in Japan. We consider these results to be useful not only for selecting good parental combinations for fruit quality or other important traits but also for determining the scale of breeding programs required to achieve specific breeding goals.

## Introduction

In the breeding of citrus (*Citrus* spp.), suitable fruit quality is critical for consumer acceptance of new cultivars. As such, fruit-quality traits including high sugar content, easy peeling, seedlessness, soft pulp, and segment softness have been the major focus of citrus breeding programs in Japan, including that of the National Agriculture and Food Research Organization (NARO) Institute of Fruit Tree and Tea Science [1].

Previous studies of citrus have shown that many important fruit-quality traits are controlled by multiple genes [2, 3]. The complex genetic background regulated by multiple genes makes it difficult to predict the segregation patterns of these traits and identify good parental combinations in citrus cross breeding. This in turn hinders the development of citrus cultivars with desirable fruit-quality traits, since genetic variability produced by good parental combinations is essential for obtaining superior genotypes [4,5].

A method for predicting the segregation of a target trait has been proposed on the basis of repeatedly measured phenotypic records in fruit breeding programs for Japanese persimmons [6] and grapes [7]. That approach describes trait segregation in F_1_ progeny derived from each parental combination as a normal distribution with the mid-parental value as the mean under the assumption of a fixed common variance for Mendelian sampling in all F_1_ families. However, the mid-parental value has poor estimation accuracy when the target trait has low heritability and there are a limited number of observations [8]; it also ignores differences in genetic variation among F_1_ families derived from different pairs of parental cultivars. These limitations make it difficult to accurately predict trait segregation for the selection of promising parental combinations in fruit breeding programs including citrus.

We recently reported an approach for selecting superior genotypes in citrus breeding programs that is based on the best linear unbiased prediction (BLUP) method [9]. That approach was able to accurately predict breeding values and estimate genetic parameters including narrow-sense heritability and genetic correlations for nine important fruit-quality traits on the basis of phenotypic records collected from the ongoing citrus breeding program at the Kuchinotsu Citrus Research Station, NARO (Nagasaki, Japan). These accurate breeding values and genetic parameters could be useful not only for selecting superior genotypes but also for predicting segregation patterns of multiple traits in F_1_ progeny and identifying good parental combinations in citrus breeding programs.

Therefore, the aims of the present study were (1) to propose a practical method for predicting multiple trait segregation patterns in an F_1_ progeny obtained by crossing parental cultivars by using breeding values of the parents and genetic parameters calculated by the BLUP method, and (2) to apply our approach to actual data from a citrus breeding population to select promising parental combinations that can produce new cultivars with high genetic performance of fruit qualities.

## Materials and methods

### Plant materials and phenotypic records

We used 111 parental cultivars and their 2011 F_1_ progeny from 126 biparental crosses obtained from the breeding program at the Kuchinotsu Citrus Research Station. The F_1_ progeny were grafted onto trifoliate orange (*Poncirus trifoliata* L.) trees during 2006–2008 (in our previous paper [9], we incorrectly reported that grafting of these materials was conducted during 2005–2007), which were planted in breeding fields at a spacing of 0.3 m within and 5 m between rows. Parental cultivars were grafted onto trifoliate orange or satsuma mandarin (*Citrus unshiu* Marcow.) interstocks in adjacent fields. Crosses were performed solely for producing commercial cultivars, and therefore no specific mating design was adopted. Five colored fruit samples were randomly harvested for immediate trait evaluation from a tree of each genotype, and nine fruit-quality traits (fruit weight, fruit skin color, fruit surface texture, peelability, flesh color, pulp firmness, segment firmness, sugar content, and acid content) were evaluated. These phenotypic records were evaluated and accumulated in the seedling selection process in our citrus breeding program. Experimental details including parental genotypes, parental combinations of F_1_ progeny, plant management, and fruit evaluation protocols have been previously reported [9].

### Breeding values and variance components

To construct a segregation model for the nine fruit-quality traits, we predicted the breeding values of all 2122 genotypes for each trait and estimated the additive genetic variance for each trait and the additive genetic covariances among all traits by using the multi-trait BLUP method. In this method, we constructed an additive genetic relationship matrix for genotypes based on the pedigree information and assumed the normality of the nine traits. The normality of each of the residuals in our multi-trait BLUP model was confirmed by visual examination. The procedures for calculating breeding values and variance components and the corresponding equations are described in our earlier study [9].

### Segregation prediction method

**Fig 1:**
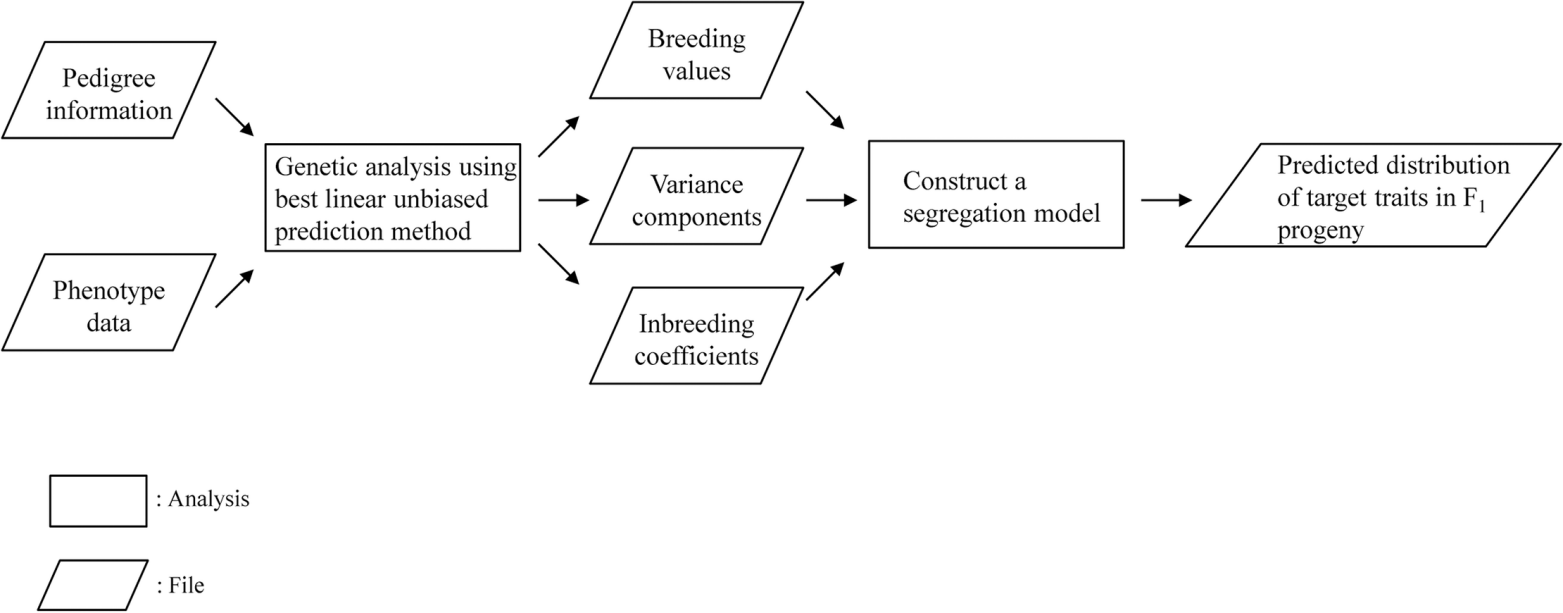
Outline for predicting target trait segregation in F_1_ progeny populations.

We constructed a segregation model for the nine fruit-quality traits in a two-generation family consisting of two parental cultivars and their F_1_ progeny as follows:

$$a_{o}∼\mathrm{MVN}(a_{mp},V),$$ (1)

where **a**_*o*_ is the vector of breeding values of F_1_ progeny for the nine fruit-quality traits and is represented as **a**_*o*_ = [*a*_1_,*a*_2_,…,*a*_9_]′ with *a*_*j*_ indicating a breeding value of the *j*th trait, **a**_*mp*_ is the vector of the midparental breeding values of the parental genotypes; and the element is calculated as (*a*_*fj*_ + *a*_*mj*_)/2, where *a*_*fj*_ and *a*_*mj*_ are the breeding values of the *j*th trait in the seed and pollen parents, respectively and **MVN** indicates a multivariate normal distribution (nine variates in this case) with **V** being the covariance matrix. We used the predicted breeding values of seed and pollen parents as *a*_*fj*_ and *a*_*mj*_, which were calculated using the BLUP method for each trait under the assumption that their F_1_ progeny was not yet produced. The covariance matrix **V**, which represents the genetic (co)variance matrix for Mendelian sampling in F_1_ progeny for the nine fruit-quality traits, is written as

$$V=\frac{1}{2}(1-\frac{F_{f}+F_{m}}{2})\left[\begin{array}{cccc} \sigma_{a1}^{2} & \sigma_{a21} & \ldots & \sigma_{an1}\\ \sigma_{a12} & \sigma_{a2}^{2} & \ldots & \sigma_{an2}\\ \vdots & \vdots & \ddots & \vdots \\ \sigma_{a1n} & \sigma_{a2n} & \ldots & \sigma_{an}^{2} \end{array}\right],$$ (2)

where *F*_*f*_ and *F*_*m*_ indicate the inbreeding coefficients of seed and pollen parents, respectively. Inbreeding coefficient refers to the fraction of homozygous loci in a genotype, which does not affect Mendelian sampling under the infinitesimal model [10]. We calculated the inbreeding coefficients from pedigree information with the R software [11] ‘nadiv’ package [12], and used these coefficients to construct the segregation model. $\sigma_{aj}^{2}$ and *σ*_*ajk*_ are elements of the additive genetic (co)variance matrix for the *j*th and *k*th traits; we incorporated their estimates ${\hat{\sigma }}_{j}^{2}$ and ${\hat{\sigma }}_{ajk}$ calculated with the restricted maximum likelihood method as a step in the BLUP method. The outline of our proposed method is shown in Fig 1.

### Validation of segregation prediction

The accuracy of the proposed method using the distribution of breeding values of F_1_ progeny (1) was evaluated in three F_1_ families: 93 progeny of ‘Tamami’ × ‘Shiranuhi’, 81 progeny of ‘Tsunonozomi’ × ‘Mihaya’, and 69 progeny of ‘Harehime’ × ‘Seinannohikari’, which were included in the 2122 genotypes described above. In each population, we compared the frequency distributions of breeding values of the nine fruit-quality traits obtained by the proposed method using (1) with sample distributions of breeding values of F_1_ progeny included in the dataset predicted by the BLUP method. The prediction of trait segregation in F_1_ progeny based on the distribution (1) was considered highly accurate if the two distributions were well coincident with one another. The coincidence between the two distributions was also evaluated by Q–Q (quantile–quantile) plots using the ‘qqplot’ function in R software.

### Selection of good parental combinations

We applied the proposed method to all possible pairs of the 2122 genotypes examined, and calculated the probabilities of obtaining progeny with characteristics superior to satsuma mandarin and ‘Shiranuhi’ (‘Kiyomi’ × ‘Nakano No. 3’ ponkan) in terms of the nine fruit-quality traits. Since the reciprocal crosses returned the same probabilities in our method, we searched for good parental combinations, including selfing among _2122_H_2_ = 2,252,503 possibilities. We set our selection criteria as fruit quality higher than that of satsuma mandarin or ‘Shiranuhi’ in nine target traits—i.e., larger fruit size, fruit skin and flesh with a deeper orange color, a smoother fruit surface texture, easier peelability, softer pulp and segments, higher sugar content, and lower acidity. The actual values of these criteria were determined from their breeding values predicted by the BLUP method described above. The probabilities of obtaining superior progeny with characteristics fulfilling the selection criteria were computed as *P* values from the multivariate normal distribution based on our proposed model, using the R software ‘mvtnorm’ package [13].

## Results

### Validation of segregation prediction

**Fig 2:**
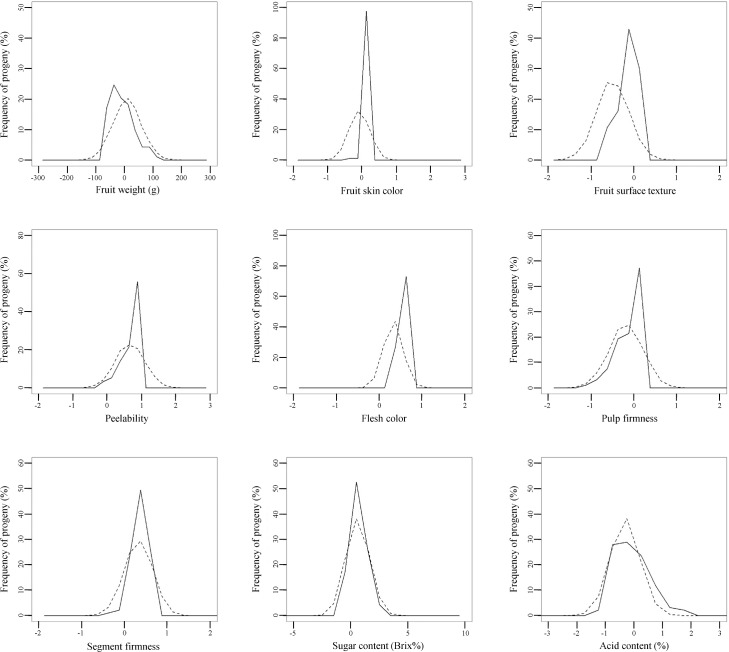
Frequency distributions of breeding values of nine fruit-quality traits in F_1_ progeny of ‘Tamami’ × ‘Shiranuhi’ predicted by the best linear unbiased prediction method (solid line) and those predicted by the proposed method for traits segregation prediction (dashed line).

**Fig 3:**
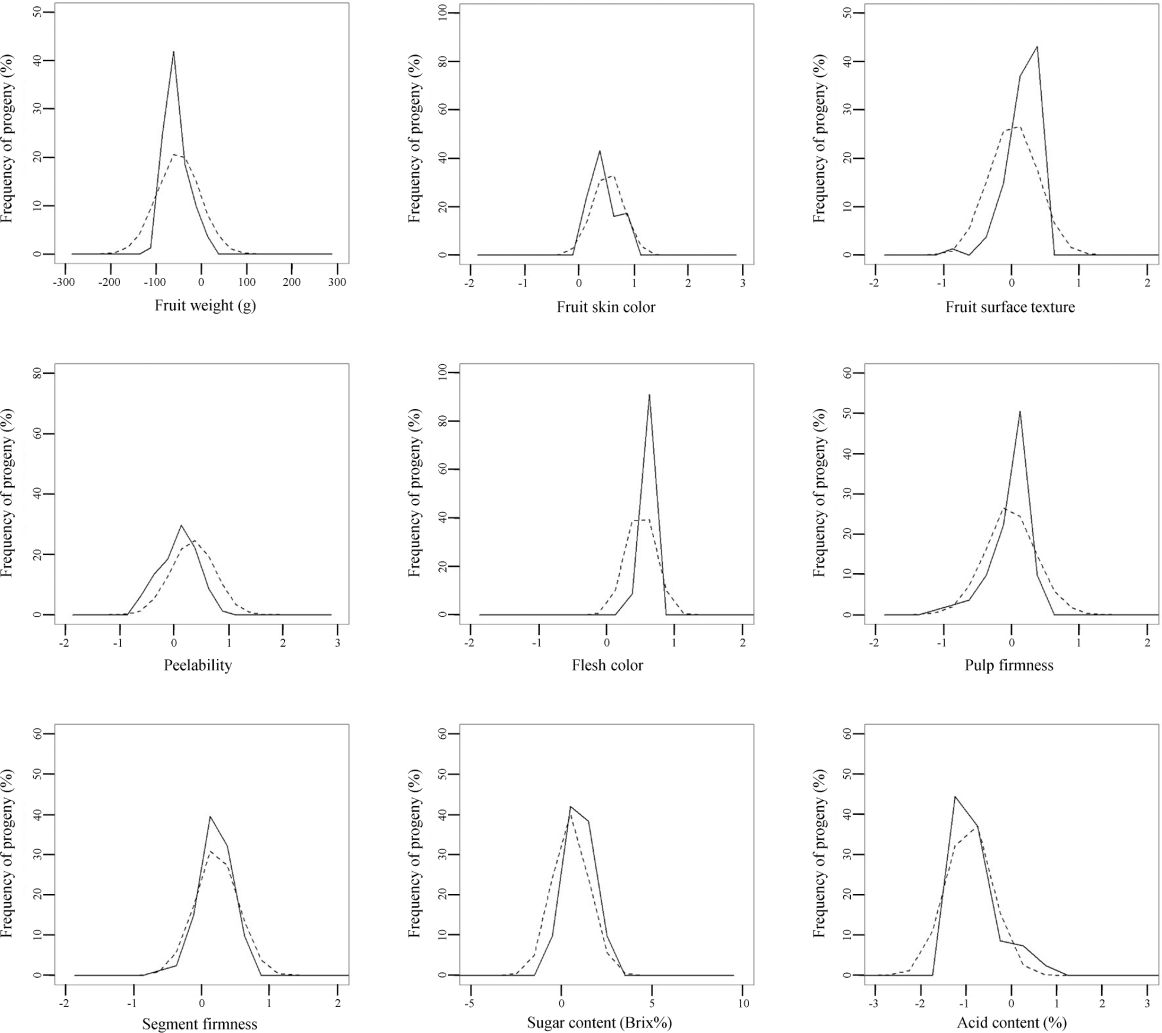
Frequency distributions of breeding values of nine fruit-quality traits in F_1_ progeny of ‘Tsunonozomi’ × ‘Mihaya’ predicted by the best linear unbiased prediction method (solid line) and those predicted by the proposed method for traits segregation prediction (dashed line).

**Fig 4:**
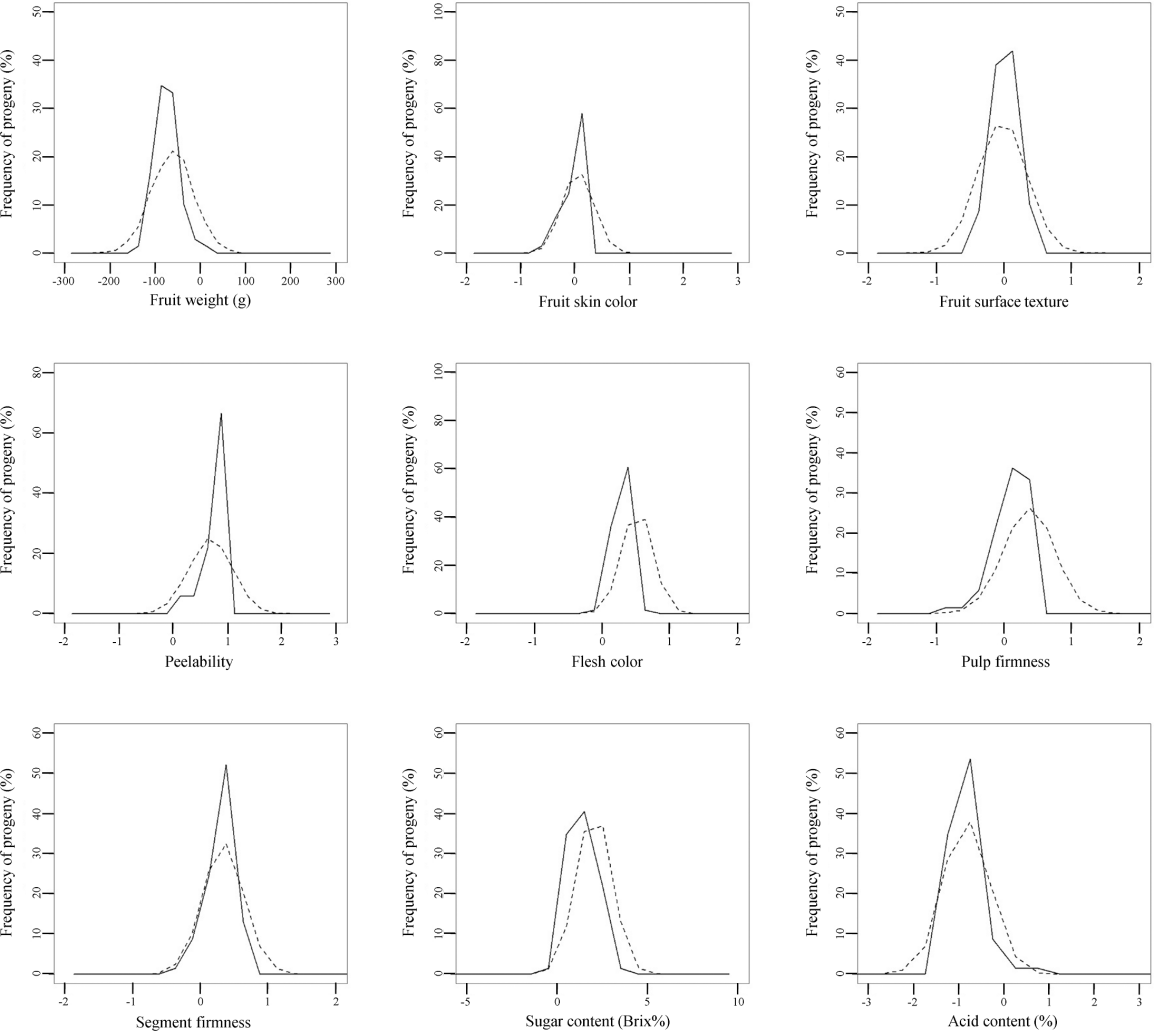
Frequency distributions of breeding values of nine fruit-quality traits in F_1_ progeny of ‘Harehime’ × ‘Seinannohikari’ predicted by the best linear unbiased prediction method (solid line) and those predicted by the proposed method for traits segregation prediction (dashed line).

The normality of the residuals, which was assumed in our multi-trait BLUP model but which we did not report in our previous paper [9], were visually confirmed (S1 Fig). Then, using the breeding values and genetic parameters of the nine fruit-quality traits calculated by the BLUP method [9], we constructed a model for trait segregation in F_1_ progeny. The accuracy of the constructed segregation model was evaluated in three F_1_ populations: 93 progeny of ‘Tamami’ × ‘Shiranuhi’, 81 progeny of ‘Tsunonozomi’ × ‘Mihaya’, and 69 progeny of ‘Harehime’ × ‘Seinannohikari’ (Figs 2–4). For fruit weight in the ‘Tamami’ × ‘Shiranuhi’ F_1_ progeny (Fig 2) and flesh color in the ‘Tsunonozomi’ × ‘Mihaya’ F_1_ progeny (Fig 3), the means of distributions of breeding values calculated from all datasets differed from the means of the distributions obtained by the constructed segregation model (1). For fruit skin color in the ‘Tamami’ × ‘Shiranuhi’ F_1_ progeny, the dispersions were somewhat different (Fig 2). However, the distributions of breeding values calculated from all datasets generally coincided with those predicted by the constructed segregation model. The coincidence between these two distributions was also confirmed by Q–Q plot (S2–S4 Figs). These results indicate that our proposed method is practical for predicting multiple trait segregation in a progeny population, and can be used to identify promising parental combinations in citrus breeding programs.

### Selection of good parental combinations

The proportion of progeny superior to satsuma mandarin or ‘Shiranuhi’ in terms of the nine fruit-quality traits was calculated for each combination among the all possible pairs of the 2122 genotypes. We ranked a total of 2,252,503 combinations of parental genotypes with the probabilities of favorable F_1_ progeny exceeding satsuma mandarin to be generated in the F_1_ populations. The best parental combination generated such F_1_ progeny with a probability of 0.0375, and in each of the best 30 parental combinations, the probabilities of favorable F_1_ progeny to be obtained were around 0.03 (Table 1). Thus, our proposed method indicates that three to four progeny superior to satsuma mandarin can be obtained from 100 F_1_ progeny derived from promising parental combinations. On the other hand, the highest probability of favorable F_1_ progeny that were superior to ‘Shiranuhi’ was 0.115, with the best 30 parental combinations having probabilities ranging from 0.08 to 0.11 (Table 2). It was easier to obtain F_1_ progeny that were superior to ‘Shiranuhi’ than to satsuma mandarin in our proposed method: that is, 8–12 F_1_ progeny superior to ‘Shiranuhi’ can be expected from 100 F_1_ progeny derived from promising parental combinations.

**Table 1:** Top 30 parental combinations with the highest probability of obtaining progeny superior to satsuma mandarin (*Citrus unshiu* Marcow.).

Parental combination		Trait^a^									
Seed parent	Pollen parent	FW	FSC	FST	PE	FC	PF	SF	SC	AC	Total^b^
**080706** (Ehime Kashi No. 28 × Seinannohikari)	**051115** (960203 × 980389)	48.4	83.0	78.8	45.4	36.1	41.3	99.8	99.5	68.7	**3.75**
**050376** (960203 × Harumi)	**070435** (Ehime Kashi No. 28 × Okitsu 56 Gou)	92.3	52.0	89.2	36.3	33.3	39.1	99.9	99.8	48.3	**3.59**
**071053** (Kuchinotsu 49 Gou × Seinannohikari)	**050376** (960203 × Harumi)	42.4	84.3	80.7	45.4	41.8	44.3	99.9	99.0	70.0	**3.57**
**080883** (Ehime Kashi No. 28 × Seinannohikari)	**051115** (960203 × 980389)	68.2	54.5	87.3	39.2	35.1	45.6	99.9	99.9	61.9	**3.53**
**050376** (960203 × Harumi)	**051115** (960203 × 980389)	92.2	81.0	83.0	40.5	32.5	34.6	99.8	98.4	50.3	**3.52**
**071012** (Kuchinotsu 49 Gou × Seinannohikari)	**080706** (Ehime Kashi No. 28 × Seinannohikari)	67.6	94.0	83.5	39.8	31.6	39.8	99.6	96.1	53.3	**3.40**
**080676** (Ehime Kashi No. 28 × Seinannohikari)	**050376** (960203 × Harumi)	94.5	99.0	68.6	34.1	34.9	32.5	98.7	89.9	63.8	**3.35**
**080688** (Ehime Kashi No. 28 × Seinannohikari)	**051115** (960203 × 980389)	89.6	88.4	67.7	37.2	31.0	41.3	99.9	99.8	52.5	**3.32**
**051115** (960203 × 980389)	**080696** (Ehime Kashi No. 28 × Seinannohikari)	74.7	88.9	85.2	34.0	37.8	39.0	99.4	98.8	64.5	**3.28**
**071012** (Kuchinotsu 49 Gou × Seinannohikari)	**080694** (Ehime Kashi No. 28 × Seinannohikari)	78.0	76.9	85.9	37.4	33.7	38.1	99.1	94.5	57.8	**3.24**
**071048** (Kuchinotsu 49 Gou × Seinannohikari)	**050376** (960203 × Harumi)	42.1	84.6	74.1	45.9	43.8	40.0	99.6	98.3	68.4	**3.22**
**070537** (satsuma mandarin × Okitsu 57 Gou)	**051115** (960203 × 980389)	76.1	53.5	81.7	36.2	33.8	35.8	99.9	99.9	59.5	**3.19**
**Ehime Kashi No. 28** (Nankou × Amakusa)	**051115** (960203 × 980389)	99.5	49.1	92.8	35.6	31.3	30.3	99.8	96.0	43.1	**3.18**
**070300** (980389 × Okitsu 56 Gou)	**050376** (960203 × Harumi)	78.9	88.7	86.7	36.1	37.7	36.7	99.0	92.2	72.3	**3.14**
**080706** (Ehime Kashi No. 28 × Seinannohikari)	**060320** (No. 1011 × Tsunonozomi)	21.3	83.4	74.1	48.8	43.6	47.4	99.9	100.0	73.8	**3.13**
**080717** (Ehime Kashi No. 28 × Seinannohikari)	**051115** (960203 × 980389)	70.2	55.9	63.2	43.4	35.3	47.4	99.8	99.4	55.0	**3.13**
**071022** (Kuchinotsu 49 Gou × Seinannohikari)	**050376** (960203 × Harumi)	73.9	78.6	89.8	29.8	36.0	46.7	99.9	98.3	48.7	**3.13**
**080706** (Ehime Kashi No. 28 × Seinannohikari)	**050376** (960203 × Harumi)	31.2	90.9	82.1	50.9	38.4	36.8	98.6	97.4	73.3	**3.10**
**050376** (960203 × Harumi)	**080694** (Ehime Kashi No. 28 × Seinannohikari)	94.1	51.7	90.3	38.6	33.2	36.7	99.9	97.6	57.1	**3.10**
**080716** (Ehime Kashi No. 28 × Seinannohikari)	**050376** (960203 × Harumi)	75.9	54.9	69.1	41.6	36.5	41.7	99.5	99.2	57.4	**3.09**
**080694** (Ehime Kashi No. 28 × Seinannohikari)	**051115** (960203 × 980389)	85.6	52.9	90.3	40.5	34.9	36.9	99.8	98.7	62.2	**3.09**
**071020** (Kuchinotsu 49 Gou × Seinannohikari)	**050376** (960203 × Harumi)	56.9	97.3	85.9	44.1	33.5	35.9	97.9	90.4	58.4	**3.07**
**080712** (Ehime Kashi No. 28 × Seinannohikari)	**051115** (960203 × 980389)	37.6	55.3	85.8	47.5	36.9	44.0	99.3	98.8	76.2	**3.07**
**080881** (Ehime Kashi No. 28 × Seinannohikari)	**071012** (Kuchinotsu 49 Gou × Seinannohikari)	61.5	82.4	79.4	44.4	34.6	40.6	99.8	98.5	64.0	**3.06**
**070395** (Ehime Kashi No. 28 × Okitsu 56 Gou)	**050376** (960203 × Harumi)	85.1	88.2	82.4	33.2	36.5	27.6	99.2	95.1	70.2	**3.01**
**080706** (Ehime Kashi No. 28 × Seinannohikari)	**080706** (Ehime Kashi No. 28 × Seinannohikari)	33.9	57.6	76.7	46.6	39.6	45.9	99.8	99.8	79.2	**2.99**
**051115** (960203 × 980389)	**051196** (Kuchinotsu 33 Gou × Okitsu 57 Gou)	68.6	98.0	78.3	39.0	37.4	34.0	98.4	91.7	66.7	**2.99**
**071048** (Kuchinotsu 49 Gou × Seinannohikari)	**051115** (960203 × 980389)	71.9	98.2	59.7	43.3	37.6	40.1	99.9	98.5	61.4	**2.99**
**071012** (Kuchinotsu 49 Gou × Seinannohikari)	**051115** (960203 × 980389)	95.8	75.1	92.7	34.1	30.2	36.0	99.7	94.1	46.1	**2.98**
**050400** (980389 × Tsunonozomi)	**050376** (960203 × Harumi)	92.4	51.8	90.0	38.1	32.7	37.2	99.8	98.5	46.8	**2.98**

*FW* fruit weight, *FSC* fruit skin color, *FST* fruit surface texture, *PE* peelability, *FC* flesh color, *PF* pulp firmness, *SF* segment firmness, *SC* sugar content, *AC* acid content

^a^ Probabilities of obtaining superior progenies in each trait

^b^ Probabilities of obtaining superior progenies in all traits

**Table 2:** Top 30 parental combinations with the highest probability of obtaining progeny superior to ‘Shiranuhi’ (‘Kiyomi’ × ‘Nakano No. 3’ ponkan).

Parental combination		Trait^a^									
Seed parent	Pollen parent	FW	FSC	FST	PE	FC	PF	SF	SC	AC	Total^b^
**080688** (Ehime Kashi No. 28 × Seinannohikari)	**060337** (No. 1011 × Tsunonozomi)	67.7	84.2	87.9	78.1	43.9	89.9	70.8	80.3	90.8	**11.53**
**Ehime Kashi No. 28** (Nankou × Amakusa)	**050391** (960203 × Harumi)	93.5	62.8	95.4	94.9	47.9	85.2	46.5	34.4	97.1	**10.94**
**080688** (Ehime Kashi No. 28 × Seinannohikari)	**050391** (960203 × Harumi)	37.9	72.7	76.8	92.4	54.8	90.1	65.3	89.4	97.5	**10.54**
**080676** (Ehime Kashi No. 28 × Seinannohikari)	**060063** (No. 1011 × Nankou)	41.0	98.4	70.0	95.3	53.7	92.5	61.8	84.5	91.0	**10.53**
**080688** (Ehime Kashi No. 28 × Seinannohikari)	**060344** (No. 1011 × Tsunonozomi)	59.9	93.7	74.9	94.7	47.0	90.2	56.4	79.8	98.0	**10.27**
**050376** (960203 × Harumi)	**080688** (Ehime Kashi No. 28 × Seinannohikari)	94.3	93.8	60.8	93.0	48.1	68.6	46.0	56.6	99.0	**10.19**
**Ehime Kashi No. 28** (Nankou × Amakusa)	**060174** (Tamami × Shiranuhi)	79.6	70.7	83.1	92.8	53.1	85.9	54.3	53.2	97.0	**10.08**
**080698** (Ehime Kashi No. 28 × Seinannohikari)	**060337** (No. 1011 × Tsunonozomi)	69.7	89.7	84.0	86.0	44.9	90.0	66.7	71.4	88.3	**9.93**
**060174** (Tamami × Shiranuhi)	**080688** (Ehime Kashi No. 28 × Seinannohikari)	83.3	93.6	67.2	90.7	49.9	89.3	63.9	49.8	83.8	**9.87**
**Ehime Kashi No. 28** (Nankou × Amakusa)	**060337** (No.1011 × Tsunonozomi)	99.5	68.4	94.4	85.3	40.5	63.0	66.5	17.1	78.9	**9.71**
**071012** (Kuchinotsu 49 Gou × Seinannohikari)	**080688** (Ehime Kashi No. 28 × Seinannohikari)	34.7	89.2	84.4	96.5	56.8	90.1	52.5	87.0	98.6	**9.36**
**060063** (No. 1011 × Nankou)	**080688** (Ehime Kashi No. 28 × Seinannohikari)	99.6	64.4	86.6	78.5	36.3	56.5	73.6	34.8	71.9	**9.32**
**Ehime Kashi No. 28** (Nankou × Amakusa)	**050376** (960203 × Harumi)	65.6	67.1	93.3	93.4	54.4	90.7	64.3	57.1	97.4	**9.24**
**080883** (Ehime Kashi No. 28 × Seinannohikari)	**050391** (960203 × Harumi)	80.9	66.2	81.3	92.8	51.4	72.9	54.0	71.1	98.9	**9.19**
**060174** (Tamami × Shiranuhi)	**080883** (Ehime Kashi No. 28 × Seinannohikari)	95.4	87.8	76.3	89.4	40.9	84.9	63.9	51.9	79.3	**8.95**
**Ehime Kashi No. 28** (Nankou × Amakusa)	**060344** (No. 1011 × Tsunonozomi)	94.2	58.8	88.7	91.5	43.6	80.8	54.7	55.3	95.1	**8.89**
**080698** (Ehime Kashi No. 28 × Seinannohikari)	**050391** (960203 × Harumi)	37.5	64.4	87.6	87.7	48.0	84.3	64.8	87.3	98.8	**8.88**
**051196** (Kuchinotsu 33 Gou × Okitsu 57 Gou)	**060337** (No. 1011 × Tsunonozomi)	85.7	72.3	62.6	90.0	56.1	67.1	55.1	71.3	98.7	**8.87**
**060063** (No. 1011 × Nankou)	**080687** (Ehime Kashi No. 28 × Seinannohikari)	81.5	66.7	68.4	88.3	48.5	81.5	62.5	73.5	94.9	**8.82**
**080883** (Ehime Kashi No. 28 × Seinannohikari)	**060337** (No.1011 × Tsunonozomi)	76.2	60.7	84.9	91.5	44.3	85.0	61.0	73.2	95.8	**8.79**
**051257** (Tsunokagayaki × Kuchinotsu 33 Gou)	**060174** (Tamami × Shiranuhi)	92.4	72.5	91.9	82.1	47.1	73.3	81.3	36.3	80.7	**8.76**
**051257** (Tsunokagayaki × Kuchinotsu 33 Gou)	**050376** (960203 × Harumi)	12.9	93.1	60.9	95.0	62.0	90.8	60.2	95.0	98.5	**8.68**
**050376** (960203 × Harumi)	**080698** (Ehime Kashi No. 28 × Seinannohikari)	43.5	90.0	65.9	95.0	59.6	75.5	53.3	94.3	99.4	**8.61**
**Ehime Kashi No. 28** (Nankou × Amakusa)	**071012** (Kuchinotsu 49 Gou × Seinannohikari)	94.9	89.9	87.9	93.3	45.0	88.6	56.2	31.8	84.9	**8.59**
**060174** (Tamami × Shiranuhi)	**051196** (Kuchinotsu 33 Gou × Okitsu 57 Gou)	37.8	87.0	71.2	93.9	52.5	86.8	60.4	94.8	97.5	**8.55**
**060344** (No. 1011 × Tsunonozomi)	**051196** (Kuchinotsu 33 Gou × Okitsu 57 Gou)	94.1	93.9	86.6	48.9	36.7	86.6	73.6	45.4	72.9	**8.50**
**060337** (No.1011 × Tsunonozomi)	**050391** (960203 × Harumi)	95.1	96.9	81.4	74.9	39.5	89.1	69.5	37.1	66.0	**8.50**
**080883** (Ehime Kashi No. 28 × Seinannohikari)	**060344** (No. 1011 × Tsunonozomi)	78.4	90.4	84.0	93.2	45.7	91.3	62.4	50.8	86.8	**8.47**
**050391** (960203 × Harumi)	**060344** (No. 1011 × Tsunonozomi)	53.4	71.6	73.0	92.0	53.2	82.9	61.8	72.7	98.3	**8.45**
**060063** (No. 1011 × Nankou)	**080686** (Ehime Kashi No. 28 × Seinannohikari)	80.2	60.1	81.1	90.8	44.0	77.5	62.0	73.5	97.2	**8.44**

*FW* fruit weight, *FSC* fruit skin color, *FST* fruit surface texture, *PE* peelability, *FC* flesh color, *PF* pulp firmness, *SF* segment firmness, *SC* sugar content, *AC* acid content

^a^ Probabilities of obtaining superior progenies in each trait

^b^ Probabilities of obtaining superior progenies in all traits

## Discussion

Selecting good parental combinations that can produce F_1_ progeny with favorable characteristics is an important determinant for the success of fruit breeding programs, including citrus. In this study, we developed a simple and practical method for predicting the segregation patterns of multiple traits in citrus. Using the proposed method, we could predict the segregation patterns of nine fruit-quality traits in a F_1_ population given the breeding values of the two parents with practical accuracy in citrus, and could identify good parental combinations that would produce progeny with fruit-quality traits exceeding predefined criteria.

The efficiency of cross-breeding in fruit crops depends largely on the accuracy of phenotypic selection for desired characteristics and the choice of good parental combinations that have high probability of obtaining superior genotypes [6]. With regard to phenotypic selection, we previously reported the potential of the BLUP method for selecting superior genotypes in a citrus breeding program [9]. With regard to selecting good parental combinations, we have proposed a segregation prediction method in this paper. Thus, we have been able to provide a practical solution for these two important problems in fruit cross-breeding. Recently, Hamilton and Kerr [14] reported an efficient computational method and an R package (“polyAinv”) for the inverse additive relationship matrix—which is essential for the BLUP method—for multiple-ploidy populations. By using their method, the BLUP method and our proposed segregation prediction method can be applied not only to diploid fruit crops, but also to multiple ploidy fruit crops including species of economic importance such as Japanese persimmons, grapes, and so on.

Another important problem in fruit breeding programs is that a huge area is needed to grow and evaluate seedlings, because of their large size [15]. Consequently, even when a good parental combination is selected, the number of progeny within the parental combination that are actually grown may often be small, and thus outstanding progeny might not be obtained. With regard to this problem, our proposed method was able to predict the probabilities of obtaining promising progeny that exceed the predefined criteria in any parental combination, as well as being able to select good parental combinations, as demonstrated in this study (Tables 1 and 2). Therefore, our proposed method can be used to determine the scale of breeding programs necessary to achieve specific breeding goals.

A limitation of our proposed method is that its accuracy for trait segregation prediction depends on the accuracy of its parameters. We used phenotypic records and pedigree information on 2122 genotypes to obtain parameters for constructing the trait segregation model. Collecting a larger dataset from multiple locations and/or for longer periods could further increase the accuracy of our prediction method. These large datasets could be established by collecting data from several citrus breeding programs, and would offer enough information for more precise prediction of trait segregation. They would also enable precise selection of prominent genotypes even in breeding programs that have been running for only a short time and have only a small amount of accumulated data by analyzing combined datasets using the BLUP method. Moreover, the set of data collected across multiple environments is applicable to the BLUP method with a genotype-by-environment interaction term (e.g., Smith et al. [16]), which may offer useful information for developing regionally adapted genotypes.

The accuracy of our proposed method also depends on the mode of inheritance of target traits. Our method considers additive polygenic effects under the assumption of an infinitesimal model [17]. However, in fruit breeding, not only additive effects but also non-additive effects (i.e., dominance and epistasis) can be utilized because superior genotypes with dominance and epistasis effects can be propagated by grafting or other asexual means. Several studies have demonstrated that the breeding values predicted by the BLUP method with an additive relationship matrix capture a large part of the dominance and epistasis effects [18, 19], and it seems justified in our case (S5 Fig); nevertheless, using a model that incorporates non-additive effects could be valuable in fruit crops (e.g., Minamikawa et al. [20]). In animal breeding, the BLUP method using pedigree information is extended to predict non-additive effects [21–23]. In these studies, one proposed model [24] may be more appropriate for fruit breeding—which typically involves inbreeding—since it can predict exact additive and dominance effects in a population with inbreeding (Narita, personal communication). Once the non-additive effects are evaluated, we can incorporate this information into our proposed method to improve the accuracy of trait segregation.

In addition to non-additive effects, the mode of inheritance of target traits would involve major genes that have significant effects on the phenotype [25]. To predict the trait segregation with a major gene, Iwanami et al. applied the segregation analysis [26] in a pedigreed apple population [27]. That study revealed the existence of a major gene controlling fruit acidity in apple, and predicted the distribution of genotypic values of F_1_ progeny in consideration of parental genotypes of the major gene. In fruit cross breeding, segregation analysis may be especially useful for a population in which molecular markers cannot be used—such as a formerly culled population—because segregation analysis requires only phenotypic records from a pedigreed population. In contrast, if molecular markers linked to QTLs are available, they can be used to predict trait segregation and to select prominent genotypes [28, 29]. When parental genotypes of major genes are available, this information can be incorporated into our proposed method, especially for predicting single-trait segregation. However, in the case of multiple traits, a trait segregation model can be more complicated because pleiotropic effects of and linkage between major genes must be taken into account.

In a recent case study of Japanese pear, a novel method was proposed for predicting the segregation of target traits based on genome-wide markers [5]. That method constructed a segregation model based on the estimated effects of each of all genome-wide markers, and can therefore predict the Mendelian sampling effects in a progeny population. Consequently, that method could be more accurate than our method. However, in ongoing fruit breeding programs where there are large phenotypic records but no molecular marker information—which is the case in most fruit breeding programs—our method offers a simple and practical way to predict segregation of target traits using large accumulated datasets. In the future, a novel method that uses the combined information derived from genotyped and non-genotyped individuals should be developed, such as the method for predicting breeding values [30, 31].

In conclusion, concerning the difficult problem of selecting parental combinations for citrus breeding, we have proposed a practical solution for selecting prominent parental combinations by using accumulated phenotypic data in ongoing citrus breeding programs. In the near future, we intend to validate our proposed method in other fruit breeding programs.

## Supporting information

S1 Fig Residuals for each trait in the multi-trait BLUP model. Frequency is shown on the vertical axis, and residuals are shown on the horizontal axis. *FW* fruit weight, *FSC* fruit skin color, *FST* fruit surface texture, *PE* peelability, *FC* flesh color, *PF* pulp firmness, *SF* segment firmness, *SC* sugar content, *AC* acid content.
(PDF)

S2 Fig Q–Q plot of frequency distributions of breeding values of nine fruit-quality traits in F1 progeny of ‘Tamami’ × ‘Shiranuhi’ predicted by the best linear unbiased prediction method (*y*-axis) and those predicted by the proposed method for traits segregation prediction (*x*-axis). Squared Mahalanobis distance was calculated from breeding values of nine fruit-quality traits, and their distributions were compared.
(PDF)

S3 Fig Q–Q plot of frequency distributions of breeding values of nine fruit-quality traits in F1 progeny of ‘Tsunonozomi’ × ‘Mihaya’ predicted by the best linear unbiased prediction method (*y*-axis) and those predicted by the proposed method for traits segregation prediction (*x*-axis). Squared Mahalanobis distance was calculated from breeding values of nine fruit-quality traits, and their distributions were compared.
(PDF)

S4 Fig Q–Q plot of frequency distributions of breeding values of nine fruit-quality traits in F1 progeny of ‘Harehime’ × ‘Seinannohikari’ predicted by the best linear unbiased prediction method (*y*-axis) and those predicted by the proposed method for traits segregation prediction (*x*-axis). Squared Mahalanobis distance was calculated from breeding values of nine fruit-quality traits, and their distributions were compared.
(PDF)

S5 Fig Comparison between additive effects (breeding value, *x*-axis) and sum of the additive and dominance effects (*y*-axis) predicted in single-trait BLUP method. Correlation coefficients between these two predicted values are shown in each trait. The computational iteration procedure was not convergent in the multi-trait model when dominance effects were included, and thus we applied the single-trait model with dominance effect. *FW* fruit weight, *FSC* fruit skin color, *FST* fruit surface texture, *PE* peelability, *FC* flesh color, *PF* pulp firmness, *SF* segment firmness, *SC* sugar content, *AC* acid content.
(PDF)
