# Supplementary figures and images for: Predicting segregation of multiple fruit-quality traits by using accumulated phenotypic records in citrus breeding
Source: PLoS One. 2018 Aug 16;13(8):e0202341. doi: 10.1371/journal.pone.0202341 (PMC6095598; doi:10.1371/journal.pone.0202341)

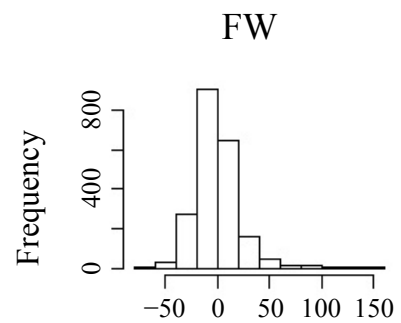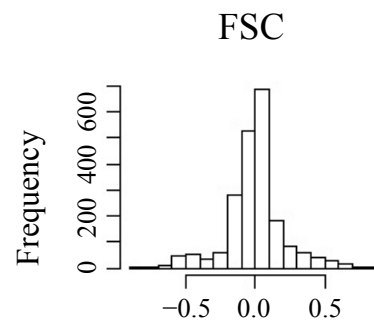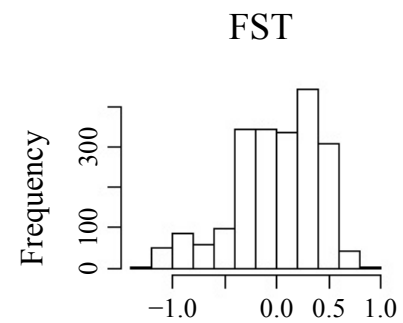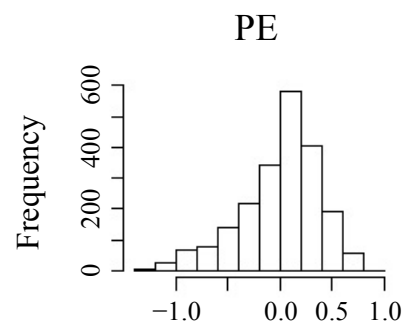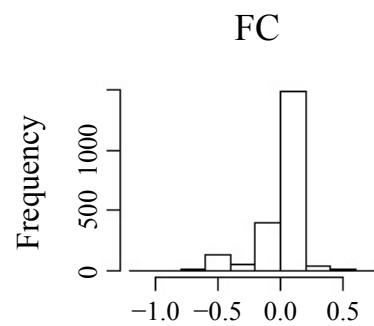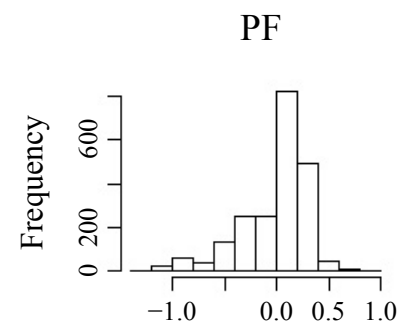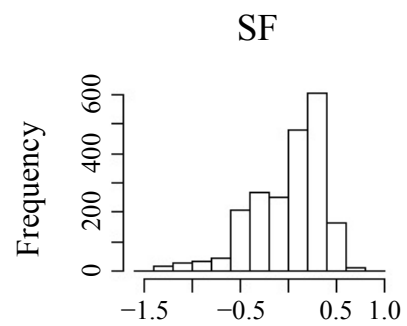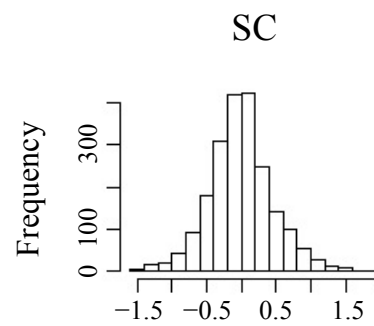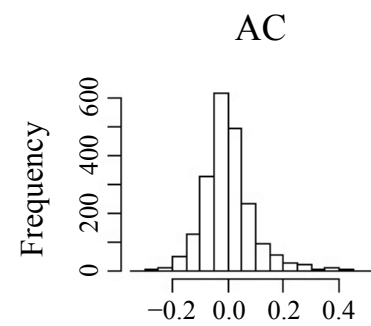

Supplement: S1 Fig — Frequency is shown on the vertical axis, and residuals are shown on the horizontal axis. FW fruit weight, FSC fruit skin color, FST fruit surface texture, PE peelability, FC flesh color, PF pulp firmness, SF segment firmness, SC sugar content, AC acid content. (PDF) [file pone.0202341.s001.pdf]

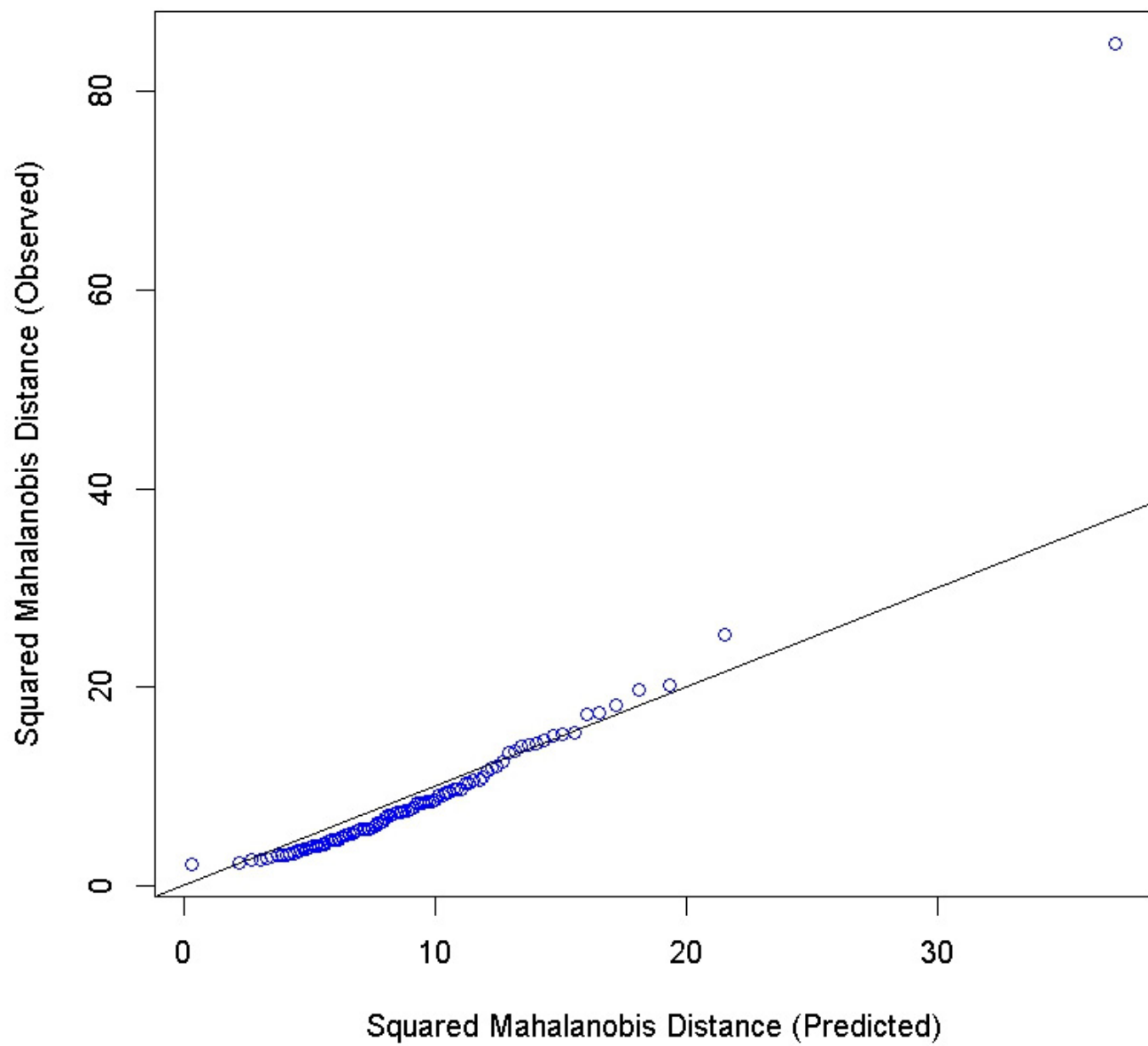

Supplement: S2 Fig — Squared Mahalanobis distance was calculated from breeding values of nine fruit-quality traits, and their distributions were compared. (PDF) [file pone.0202341.s002.pdf]

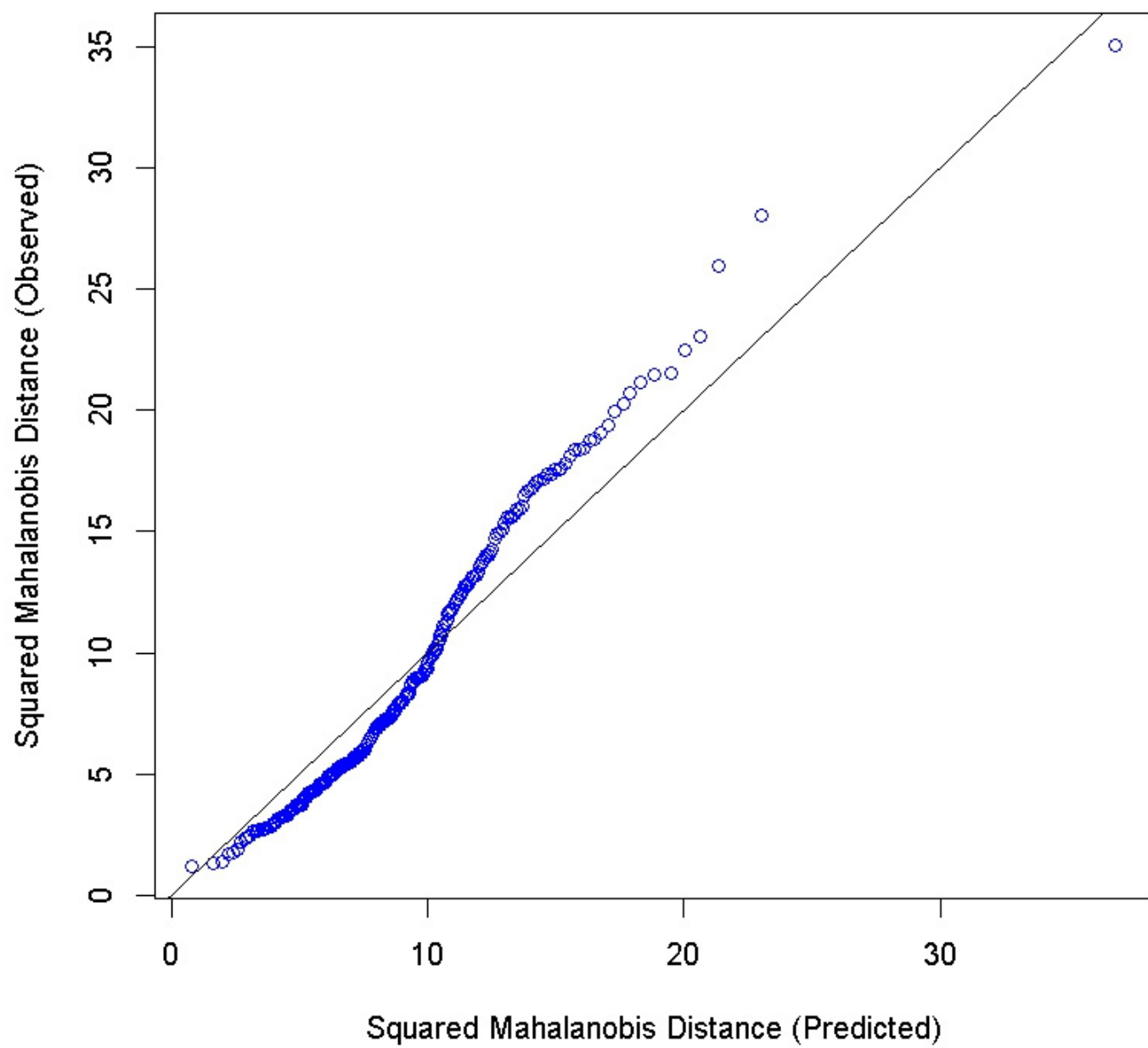

Supplement: S3 Fig — Squared Mahalanobis distance was calculated from breeding values of nine fruit-quality traits, and their distributions were compared. (PDF) [file pone.0202341.s003.pdf]

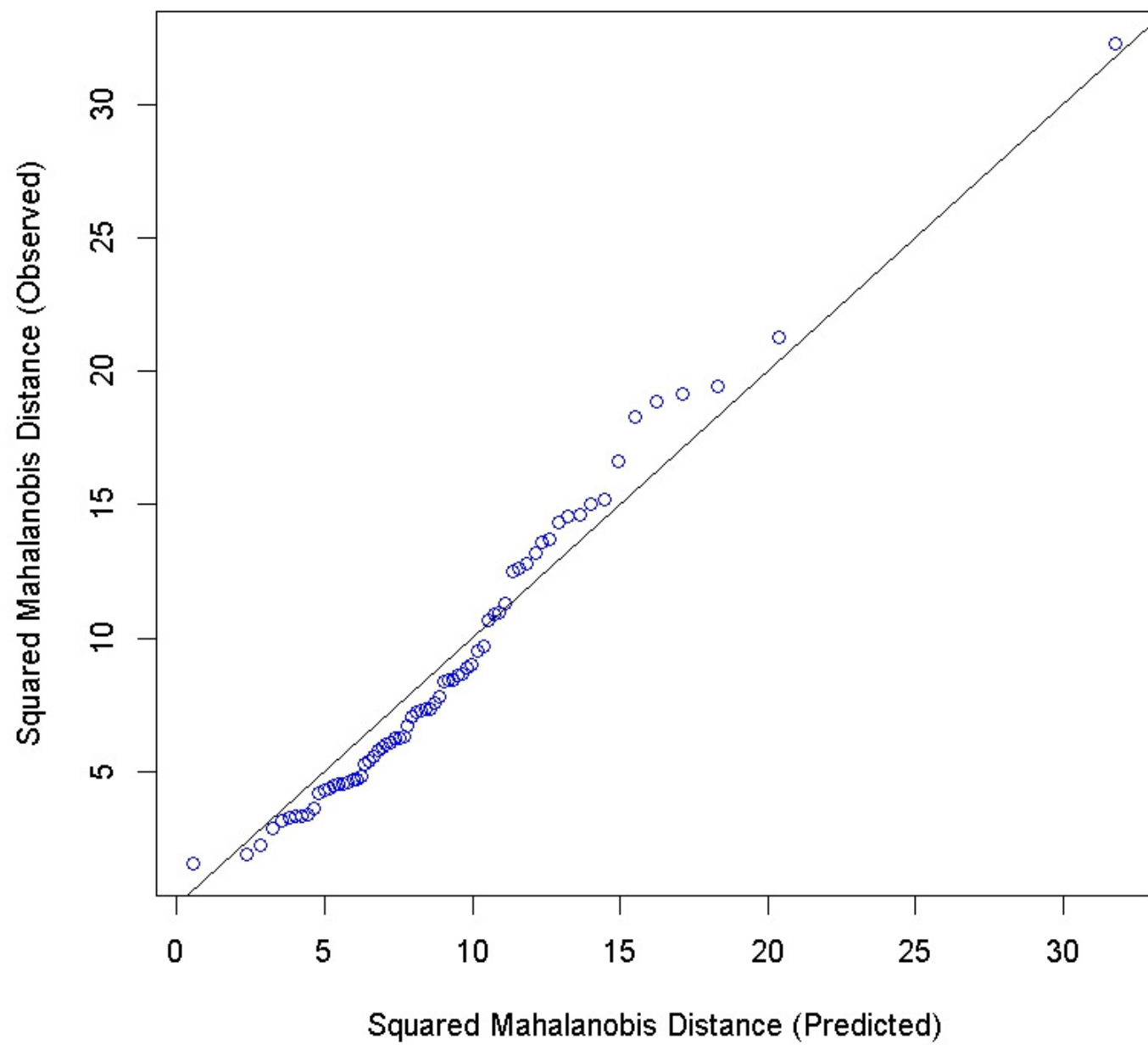

Supplement: S4 Fig — Squared Mahalanobis distance was calculated from breeding values of nine fruit-quality traits, and their distributions were compared. (PDF) [file pone.0202341.s004.pdf]

Additive plus dominance effect

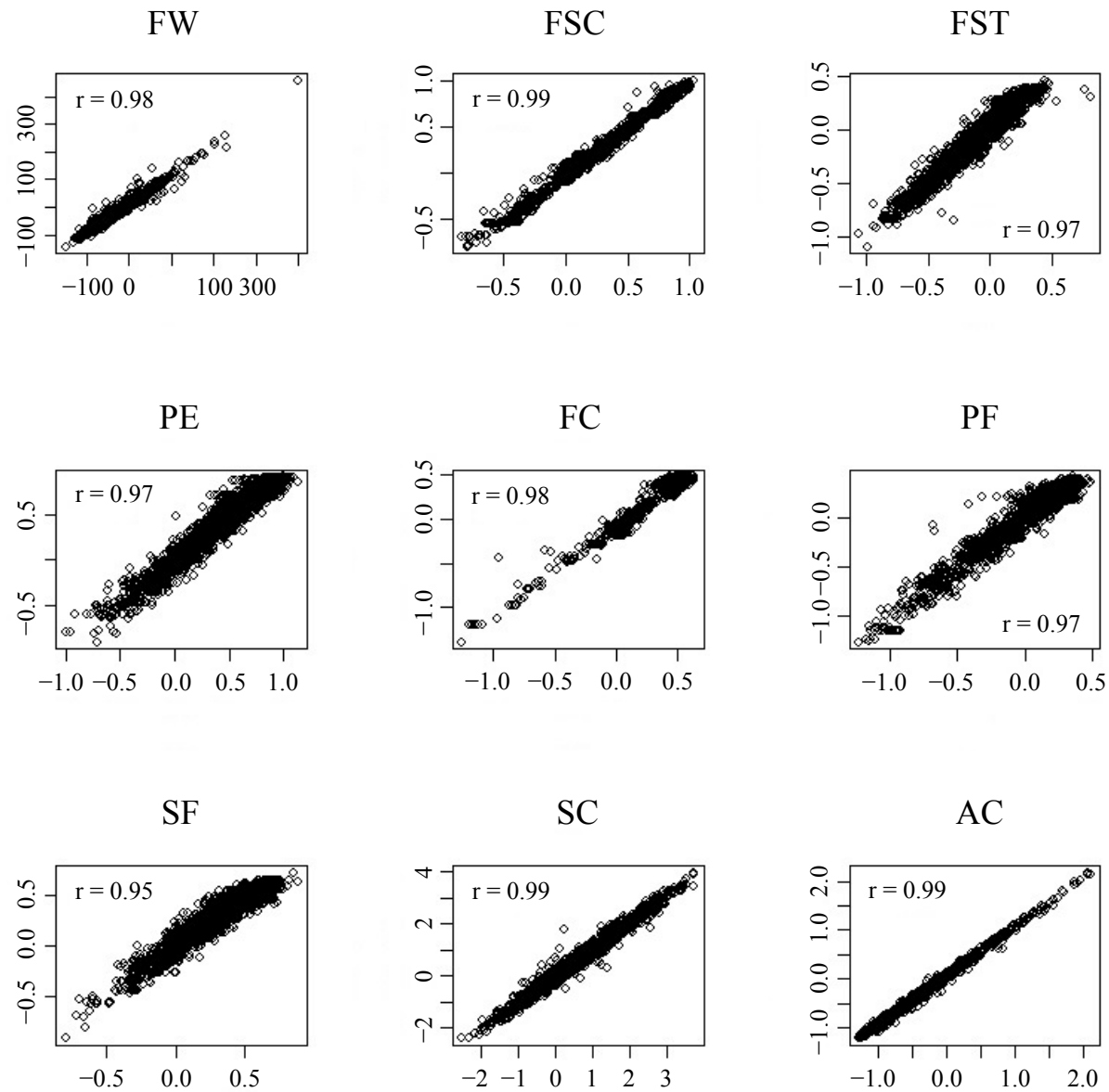

Additive effect (breeding value)

Supplement: S5 Fig — Correlation coefficients between these two predicted values are shown in each trait. The computational iteration procedure was not convergent in the multi-trait model when dominance effects were included, and thus we applied the single-trait model with dominance effect. FW fruit weight, FSC fruit skin color, FST fruit surface texture, PE peelability, FC flesh color, PF pulp firmness, SF segment firmness, SC sugar content, AC acid content. (PDF) [file pone.0202341.s005.pdf]
